# Potential ‘accelerator’ and ‘brake’ regulation of theanine biosynthesis in tea plant (*Camellia sinensis*)

**DOI:** 10.1093/hr/uhac169

**Published:** 2022-07-25

**Authors:** Jiayi Guo, Biying Zhu, Ying Chen, Shijia Lin, Siming Qiao, Fuli Ma, Shihua Zhang, Tianyuan Yang, Qi Chen, Linlin Liu, Zhaoliang Zhang, Xiaochun Wan

**Affiliations:** State Key Laboratory of Tea Plant Biology and Utilization, Anhui Agricultural University, Hefei, Anhui 230036, China; State Key Laboratory of Tea Plant Biology and Utilization, Anhui Agricultural University, Hefei, Anhui 230036, China; State Key Laboratory of Tea Plant Biology and Utilization, Anhui Agricultural University, Hefei, Anhui 230036, China; State Key Laboratory of Tea Plant Biology and Utilization, Anhui Agricultural University, Hefei, Anhui 230036, China; State Key Laboratory of Tea Plant Biology and Utilization, Anhui Agricultural University, Hefei, Anhui 230036, China; College of Horticulture, Anhui Agricultural University, Hefei, Anhui 230036, China; School of Life Science and Health, Wuhan University of Science and Technology, Wuhan, Hubei 430072, China; State Key Laboratory of Tea Plant Biology and Utilization, Anhui Agricultural University, Hefei, Anhui 230036, China; State Key Laboratory of Tea Plant Biology and Utilization, Anhui Agricultural University, Hefei, Anhui 230036, China; State Key Laboratory of Tea Plant Biology and Utilization, Anhui Agricultural University, Hefei, Anhui 230036, China; State Key Laboratory of Tea Plant Biology and Utilization, Anhui Agricultural University, Hefei, Anhui 230036, China; State Key Laboratory of Tea Plant Biology and Utilization, Anhui Agricultural University, Hefei, Anhui 230036, China

Dear Editor,

Theanine, one of the most important tea quality components, confers the ‘umami’ taste and anti-stress benefits of tea infusion [1]. Theanine is a unique non-proteinaceous amino acid solely accumulating to a high level in tea plants, and is primarily synthesized in the roots from glutamate (Glu) and ethylamine (EA) by theanine synthetase (CsTSI) [1, 2]. The high EA availability was suggested to be why only tea plants can synthesize large amounts of theanine [3]. Our recent study further indicated that EA contents in the roots of various tea plant cultivars were highly correlated with the theanine contents in the roots [4]. EA is synthesized from alanine by alanine decarboxylase CsAlaDC [4, 5]. Further experiments suggested that the CsAlaDC and CsTSI work in tandem to determine theanine biosynthesis in tea plants [4]. Interestingly, the expression of *CsTSI* in the roots of different tea plant cultivars was not obviously correlated with the theanine contents; while the expression of *CsAlaDC* was shown to be highly correlated with both EA contents and theanine contents in the roots [4]. These results indicated that the regulation of *CsAlaDC* expression is critical for adjusting the activity of theanine synthesis in tea plants. However, the mechanism underlying the regulation of *CsAlaDC* expression is largely unknown in tea plants. Uncovering the mechanism will be critical for improving theanine biosynthesis in tea plants.

**Figure 1:**
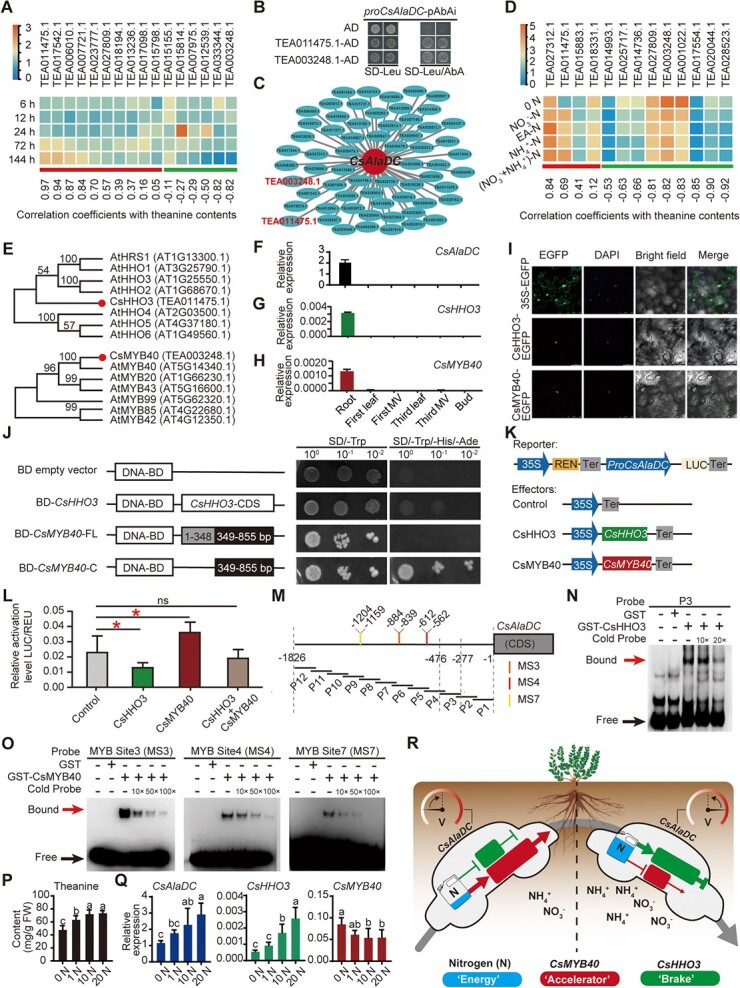
*CsHHO3* and *CsMYB40* play opposite regulatory roles in *CsAlaDC* expression to regulate theanine biosynthesis in tea plants. (**A**) Expression of 16 theanine-associated genes encoding transcription factors (TFs) under ethylamine (EA) treatment for 6, 12, 24, 72, and 144 h, and the correlation coefficients with the theanine contents, in the roots. (**B**) Yeast one-hybrid assay to test the binding of TEA011475.1 and TEA003248.1 to *CsAlaDC* promoter. (**C**) Bipartite network of TFs-*CsAlaDC* gene co-expression network. (**D**) Expression of 13 root-specific TF encoding genes under the treatment of 0 N, 1.43 mM NO_3_^+^-N, 1.43 mM EA-N, 1.43 mM NH_4_^+^-N, or 0.36 mM NO_3_^+^ plus 1.07 mM NH_4_^+^-N ([NO_3_^+^ + NH_4_^+^]-N) for 10 days, and the Pearson correlation coefficients (PCC-value) with the theanine contents, in the roots. (**E**) Phylogenetic analysis of TEA011475.1 and TEA003248.1 with their homologues in *Arabidopsis thaliana*. (**F–H**) Tissue-specific expression patterns of *CsAlaDC*, *CsHHO3* and *CsMYB40*. The expression level was determined by real-time quantitative RT-PCR. First MV, major vein in the first leaf; third MV, major vein in the third leaf. MV was obtained by separated the major vein from the leaf. (**I**) Subcellular localization of CsHHO3 and CsMYB40. (**J**) Transactivition assay of full-length or truncations of CsHHO3 and CsMYB40 in yeast. (**K**) A schematic illustration of the effector and reporter constructs in transcriptional activity of the *CsAlaDC* promoter. (**L**) Regulation of the transient transcriptional activity of the *CsAlaDC* promoter by CsHHO3 and CsMYB40 in tobacco epidermal cell. (**M**) Schematic representation of the fragments used for transcription factor binding site screening. (**N**, **O**) EMSA assay of CsHHO3 (**N**) and CsMYB40 (**O**) to exam the binding to different regions of *CsAlaDC* promoter. Cold probe represents the putative binding motif without biotin labeling. (**P**) Theanine contents in the roots of tea plants under 0 N, 1 N, 10 N, and 20 N treatments. (**Q**) The relative expression of *CsAlaDC*, *CsHHO3*, and *CsMYB40* under the four nitrogen treatments. (**R**) The proposed ‘accelerator’ and ‘brake’ regulation model of theanine biosynthesis in tea plants. The arrows and bar-ended lines represent promotion and inhibition, respectively. Thick and thin lines mean strong and weak effects, respectively.

In our previous study, using weighted genome-wide co-expression network assay (WGCNA), we identified 16 MYB transcription factors which were highly correlated with the expression of theanine pathway genes under various conditions [6]. Within these 16 genes encoding MYB transcription factors, TEA011475.1 and TEA003248.1 were up- and down-regulated, respectively, by nitrate, ammonium, and EA treatments [6], suggested these two genes might play important and divergent roles in regulating theanine biosynthesis. Consistently, further experiments showed that, within the 16 genes, the expression of TEA011475.1 and TEA003248.1 showed the highest positive and negative correlation with theanine contents in the roots, respectively, under 20 EA mM treatment for 6 h, 12 h, 1 d, 3 d, and 6 d [6] (Fig. 1A). We hypothesized that these two MYB transcription factors regulate the expression of *CsAlaDC*. To verify our hypothesis, we first performed yeast one-hybrid assay, and the result suggested that TEA011475.1 and TEA003248.1 bound to the promoter of *CsAlaDC* (Fig. 1B).

In our other previous study, we performed transcriptomic analyses on the roots of tea plants treated with 0 N, NO_3_^+^-N (1.43 mM), EA-N (1.43 mM), NH_4_^+^-N (1.43 mM), or NO_3_^+^ plus NH_4_^+^-N (1.43 mM) for 10 days [7]. The transcriptomic data was used to construct the gene co-expression network of genes encoding transcription factors and *CsAlaDC*, with *CsAlaDC* as the hub gene, to identify potential transcription factors regulating *CsAlaDC* transcription (Fig. 1C). These genes encoding transcription factors from the gene co-expression network were further narrowed down by selecting root-specifically expressed genes. In this way, 13 *CsAlaDC*-associated and root-specific genes encoding transcription factors were obtained. Interestingly, both TEA011475.1 and TEA003248.1 were also included in these 13 genes. Again, the expression of TEA011475.1 and TEA003248.1 was shown to be respectively induced and repressed by N, and highly correlated with the theanine contents in the opposite manner (Fig. 1D). These results further suggested critical and different roles of TEA011475.1 and TEA003248.1 in *CsAlaDC* expression and theanine biosynthesis.

According to the results of phylogenetic analysis, we named TEA011475.1 and TEA003248.1 as *CsHHO3* and *CsMYB40*, respectively, following the names of their homologues in model plant *Arabidopsis* (Fig. 1E). The tissue-specific expression showed that both *CsHHO3* and *CsMYB40* specifically expressed in the roots, as well as *CsAlaDC* (Fig. 1F–H). Consistent with the prediction that CsHHO3 and CsMYB40 are transcription factors, CsHHO3 and CsMYB40 both localized in the nucleus (Fig. 1I). The results of transcription activation assay suggested that CsHHO3 did not have transcription activation activity, while the predicted transcription activation domain of CsMYB40 had transcription activation activity, although the full length of CsMYB40 did not (Fig. 1J). This is consistent with the homologue of CsHHO3 and CsMYB40 in *Arabidopsis* being transcription repressor and activator, respectively [8, 9].

To test the regulatory role of CsHHO3 and CsMYB40 in *CsAlaDC* expression, we next conducted a transactivation assay in tobacco leaves using *CsAlaDC* promoter-driven Luciferace (*CsAlaDC* pro:Luc) as a reporter (Fig. 1K). The results showed that when co-transformation 35S:*CsHHO3* and *CsAlaDC* pro:Luc, the expression of CsHHO3 significantly repressed the activity of *CsAlaDC* promoter (Fig. 1L). In contrast, the expression of CsMYB40 significantly activated the activity of *CsAlaDC* promoter. Moreover, when co-expressed 35S:*CsHHO3* and 35S:*CsMYB40* (CsHHO3 + CsMYB40) with *CsAlaDC* pro:Luc, the activity of *CsAlaDC* promoter was not obviously different from the no-effector control (*CsAlaDC* pro:Luc). These results demonstrated that CsHHO3 represses, and CsMYB40 activates, the expression of *CsAlaDC in planta*.

To investigate whether CsHHO3 and CsMYB40 directly bind to the *CsAlaDC* promoter, we conducted an electrophoretic mobility shift assay (EMSA) with recombinant CsHHO3 and CsMYB40 proteins. The binding sites were first screened by testing the binding of CsHHO3 and CsMYB40 with the 12 fragments (200 bp each) of the promoter (Fig. 1M). Through this screening, we found CsHHO3 bound to the proximal region (−476 to −277) of *CsAlaDC* promoter, and CsMYB40 bound to three regions (−427 to −626, −727 to –926, and –1027 to –1226) (Fig. 1N, O). By looking for *cis*-regulatory elements recognized by MYB TFs, the CsMYB40 binding regions were further narrowed to −612 to −562, −884 to −839, and −1204 to −1159. These regions include the MYB-core consensus [C/T]NGTT[G/T] identified in protein-binding microarray [10]. These results indicated that CsHHO3 and CsMYB40 can directly bind to different regions of the *CsAlaDC* promoter.

To further investigate the regulatory roles of CsHHO3 and CsMYB40 in *CsAlaDC* expression in response to N levels, we grew tea plants in hydroponic solution with various concentrations of N (0 N, 1 N, 10 N, and 20 N; 1 N was 0.72 mM NO_3_^+^-N plus NH_4_^+^-N) for 18 days. Compared with the 0 N, 1 N, and 10 N significantly increased theanine contents in the roots (Fig. 1P); however, 20 N did not further increase the content compared with 10 N. The changes of expression of *CsAlaDC* in the roots showed a similar pattern as that of theanine contents (Fig. 1Q). Under the same conditions, the expression of *CsHHO3* was gradually induced by increased concentrations of N, while that of *CsMYB40* was repressed (Fig. 1Q). These results implied that CsHHO3, the potential repressor of *CsAlaDC* transcription, is induced by higher levels of N; however, CsMYB40, the potential activator of *CsAlaDC* transcription, is repressed by higher levels of N. Therefore, CsHHO3 and CsMYB40 likely work in tandem to maintain the expression level of *CsAlaDC* within a certain range in response to N levels, to keep the robustness of theanine biosynthesis. This is just like driving a vehicle on an expressway, hitting the accelerator when the vehicle slows down, and hitting the brake when the speed is too high, to keep the vehicle running at a high but safe speed (Fig. 1R).

In brief, we identified CsHHO3 and CsMYB40 as a repressor and an activator of *CsAlaDC* transcription, and their opposite expression patterns and regulatory roles likely maintain the robustness of ethylamine and theanine biosynthesis in response to nitrogen fluctuations. This study demonstrates a potential ‘accelerator’ and ‘brake’ regulation mode of theanine biosynthesis in tea plants (Fig. 1R). In future, we will further knock down and overexpress *CsHHO3* and *CsMYB40* to verify the regulation mode when the gene transformation system is well established in tea plants.

## Acknowledgments

This work was supported by grants from the National Natural Science Foundation of China (32072624, U21A20231), the National Key R&D Program of China (2021YFD1601101, 2018YFD1000601) and the Base of Introducing Talents for Tea Plant Biology and Quality Chemistry (D20026).

## Author contributions

Z.Z., X.W., J.G., Q.C., and L.L. conceived the study and designed the experiments; J.G., B.Z., Y.C., S.L., S.Q., and F.M. carried out the experiments; J.G., B.Z., S.L., and T.Y. analysed data and organize figures; J.G., Z.Z. and X.W. wrote the manuscript. All authors reviewed and approved the final manuscript.

## Data availability

Data are available upon request to the corresponding author.

## Conflict of interest

The authors declare that they have no conflict of interest.
